# Synchronous Improvement of Mechanical and Room-Temperature Damping Performance in Light-Weight Polyurethane Composites by a Simple Carbon-Coating Strategy

**DOI:** 10.3390/polym17152115

**Published:** 2025-07-31

**Authors:** Qitan Zheng, Zhongzheng Zhu, Junyi Yao, Qinyu Sun, Qunfu Fan, Hezhou Liu, Qiuxia Dong, Hua Li

**Affiliations:** 1 State Key Laboratory of Metal Matrix Composites, School of Materials Science and Engineering, Shanghai Jiao Tong University, Shanghai 200240, China; zhengqitan@sjtu.edu.cn (Q.Z.); zzzjtu@sjtu.edu.cn (Z.Z.); yjy2000@sjtu.edu.cn (J.Y.); sunnyqy@sjtu.edu.cn (Q.S.); fanqunfu@sjtu.edu.cn (Q.F.); hzhliu@sjtu.edu.cn (H.L.); 2 School of Environmental Science and Engineering, Shanghai Jiao Tong University, Shanghai 200240, China; 3 Inner Mongolia Research Institute, Shanghai Jiao Tong University, Hohhot 010010, China

**Keywords:** polyurethane elastomers, carbon coating, damping properties, mechanical properties, interface damping

## Abstract

In order to address vibration and noise challenges in modern industry while satisfying the lightweighting requirements for aerospace and transportation applications, the development of polymer elastomers integrating both lightweight and high-damping properties holds substantial significance. This study developed polyurethane (PU) with optimized damping and mechanical properties at room temperature through monomer composition optimization. Hollow glass microspheres (HGMs) were introduced into the PU matrix to increase stiffness and reduce density, though this resulted in decreased tensile strength (Rm) and loss factor (tanδ). To further improve mechanical and damping properties, we applied a carbon coating to the surface of the HGMs to optimize the interface between the HGMs and the PU matrix, and systematically investigated the energy dissipation and load-bearing behavior of PU composites. The effect of enhanced interface damping of HGM@C/PU resulted in broadening of the effective damping temperature range (tanδ ≥ 0.3) and higher maximum loss factor (tanδ_max_) compared to HGM/PU at equivalent filler loading. The tensile and dynamic properties significantly improved due to optimized interfacial adhesion. In PU composites reinforced with 10 wt% HGM and HGM@C, a 46.8% improvement in Rm and 11.0% improvement in tanδ_max_ occurred after carbon coating. According to acoustic testing, average transmission loss of HGM/PU and HGM@C/PU with the same filler content showed a difference of 0.3–0.5 dB in 500–6300 Hz, confirming that the hollow structure of the HGMs was preserved during carbon coating.

## 1. Introduction

Industrial machinery inevitably generates vibration and noise during operation, not only compromising equipment precision and service life, but also damaging human hearing and mental health [1,2]. To solve this problem, vibration-damping materials, especially polymer viscoelastic damping materials, have been widely used in engineering. Rubbers and some kinds of polyurethanes (PU) possess considerable damping properties [3,4,5,6]. However, most rubbers require a hot-pressing process, limiting their applicability in situations requiring complex shapes and structures. Developing castable polyurethane elastomers is one way to overcome the drawbacks of rubbers fabricated by hot pressing [7,8]. Some strategies have been proposed to improve the damping properties of PU, such as introducing dangling chains [9,10], incorporating dynamic bonds [11,12,13] or forming polymer interpenetrating networks [14,15], so that the maximum loss factor (tanδ_max_) or the effective damping temperature range (EDTR: tanδ ≥ 0.3) could be improved. However, some novel monomers that could develop high-quality PUs are cost-prohibitive for mass production, or require a large number of organic solvents during the synthetic process, leading to difficulty in controlling bubbles in PU during the solvent evaporation and weakening its mechanical properties. In addition, weight reduction has become a critical focus in areas such as aerospace and vehicle engineering, and most damping materials operate at room temperature in these fields [16]. Therefore, developing low-cost, solvent-free, and light-weight castable PUs with balanced mechanical and room temperature damping performance remains essential.

Hollow glass microspheres (HGMs) have emerged as effective fillers for density reduction [17,18,19]. Due to their unique hollow structure, the stacking density of HGMs is very low compared with conventional fillers, and the volume density of HGM-filled composites can be flexibly modulated by adjusting HGM particle size and content. According to previous research, spherical HGMs can also enhance polymer rigidity and compression resistance [20,21], concurrently improving the abrasion performance [22]. Moreover, the confined air within cavities attenuates sound waves, boosting sound insulation properties [23], and composites with HGMs are regarded as effective sound insulation materials [24,25]. However, excessive HGM loading reduces damping performance [26,27,28], necessitating careful balancing between density reduction and damping efficacy. Previous studies have demonstrated that incorporating carbon materials like carbon nanofibers [29], graphene [30], and carbon nanotubes [31] improved the mechanical properties of high-volume-ratio HGM composites. These carbon additives also improve damping performance by enhancing interfacial thermal conduction losses between polymer chains and fillers [32,33,34,35,36]. However, under normal circumstances, direct blending of carbon fillers and HGMs with resin increases the polymer matrix viscosity, reducing resin fluidity to levels incompatible with casting processes. In addition, the uniform dispersion of carbon nanofillers in the polymer matrix is notoriously prone to aggregation. Given that HGMs possess a spherical geometry with minimal surface energy, they can reduce the tendency of agglomeration of carbon nanofillers. Consequently, loading carbon materials on HGM surface leverages the inherently superior dispersion characteristics of HGMs, thereby mitigating aggregation issues in the composite system [37,38]. However, the damping and mechanical properties of such carbon-coated HGM-reinforced composites have not been systematically investigated.

In this study, PU with balanced room temperature damping and mechanical properties was designed by optimizing monomer types and ratios. Then, a facile carbon modification route was introduced. Polydopamine (PDA) was self-assembled on the surface of HGMs and further carbonized to construct a rough carbon-coated layer. Carbon-coated HGMs (HGM@C) were then incorporated into the optimized PU matrix at varying mass ratios. HGM@C/PU composites were fabricated via a simple pouring process. Mechanical properties, damping behavior, and acoustic performance of HGM@C/PU composites were systematically evaluated. Comparative analysis with uncoated HGM/PU composites demonstrated the specific contribution of the carbon coating. For instance, in PU composites reinforced with 10 wt% HGM and HGM@C, tensile strength increased from 13.9 MPa to 20.4 MPa after carbon coating. Concurrently, tanδ_max_ rose from 0.536 to 0.595, while the tanδ area (TA value) increased from 14.741 to 17.817, confirming improved damping and mechanical properties resulting from the carbon coating.

## 2. Experimental Section

### 2.1. Materials

Dicyclohexylmethane diisocyanate (HMDI, AR) was purchased from Titan Technology Co., Ltd. (Shanghai, China); polytetrahydrofuran ether diol (PTMG, M = 650, 1000 and 2000 g/mol, technicalgrade) was purchased from Yantai Wanhua Polyurethane Co., Ltd. (Yantai, China); and dimethylthiotoluene diamine (DMTDA, AR), Glycerol (GL, AR), dibutyltin dilaurate (DBTDL, AR), dopamine hydrochloride (DA·HCl, AR), and tri(hydroxymethyl) aminomethane (Tris, AR) were obtained from Aladdin Biochemical Technology Co., Ltd. (Shanghai, China). Hollow glass microbeads (HGMs, density: 0.39–0.42 g/cm^3^, diameter: 40–70 μm) were purchased from Zhengzhou Shengwright Hollow Microbeads New Material Co., Ltd. (Zhengzhou, China).

### 2.2. Preparation of HGM@C/PU Composites

Synthesis of PU elastomer: Predetermined quantities of PTMG and GL were dehydrated at 120 °C for 2 h to achieve the removal of the water content in the polyol. HMDI and PTMG were reacted at 80 °C for 2 h under continuous stirring. Then, DMTDA was introduced into the prepolymer mixture and stirred at 80 °C for 1 h to complete chain extension. After that, GL was incorporated as a crosslinking agent and homogenized with the prepolymer at 80 °C for 30 min. DBTDL (0.005 wt% relative to PU mass) was added with 3 min of stirring. After vacuum defoaming for 10 min, PU was cast into a metal mold and kept in the oven at 80 °C for 24 h. The mole ratios of HMDI, PTMG, DMTDA, and GL for distinct PU elastomers are detailed in Table 1.

**Figure 1:**
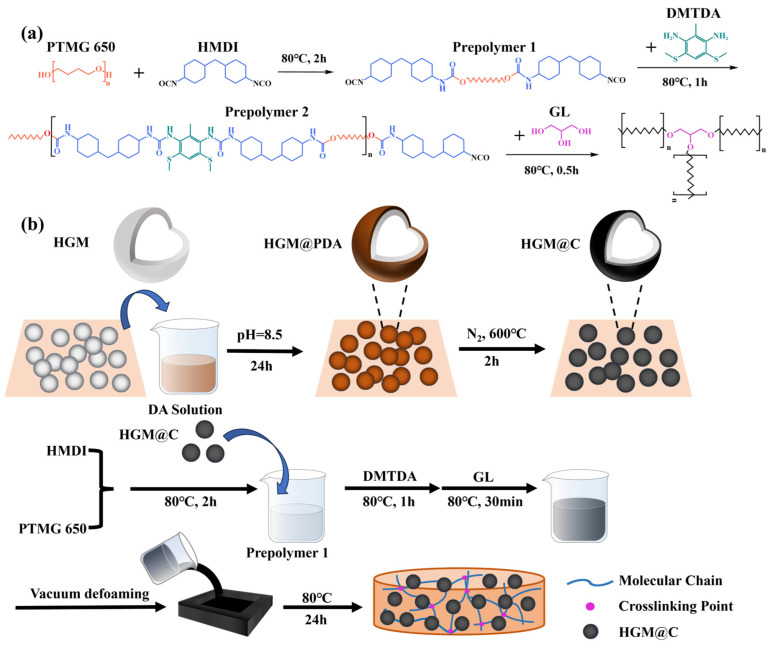
Synthesis of (**a**) PU elastomer; (**b**) HGM@C and HGM@C/PU composites.

Synthesis of HGM@C: First, 6.5 g of Tris was dissolved in 500 mL of deionized water to stabilize the pH at about 8.5. After 5 g of dopamine hydrochloride (DA·HCl) was added to the solution under stirring for 5 min, 10 g of HGMs were introduced, and a mechanical agitator was used to stir at 250 rpm for 24 h to ensure complete polydopamine (PDA) coating on HGMs. Then, HGM@PDA was collected via vacuum-assisted filtration, followed by drying at 60 °C for 24 h and sieving through a 100-mesh screen. To carbonize PDA, HGM@PDA was placed in a tube furnace under a nitrogen atmosphere, while the temperature was increased to 600 °C with a heating rate of 5 °C/min and held for 2 h. After sifting, HGM@C was finally obtained. The whole synthetic route of HGM@C is shown in Figure 1b. As mass reduction occurred during the transfer process and mesh screening, about 9.3 g of HGM@C was collected.

Preparation of HGM@C/PU: HGM@C at various mass fractions (5 wt%, 10 wt%, 15 wt%) was mechanically blended with PU prepolymer (HMDI + PTMG) for 5 min. Subsequent processing followed identical procedures as described previously to obtain HGM@C/PU elastomers as illustrated in Figure 1b. The obtained composites were denoted as PU-5C, PU-10C, and PU-15C based on HGM@C mass content. For comparison, original unmodified HGM at equivalent loadings (5 wt%, 10 wt%, 15 wt%) was incorporated into PU, and the samples were designated as PU-5, PU-10, and PU-15, respectively.

### 2.3. Characterization

Materials characterization: The morphology of HGM@C and PU composites was observed by scanning electron microscopy (SEM, Hitachi S-4800, Hitachi, Japan). X-ray diffraction analysis (XRD, Rigaku D, Japan) characterized the internal crystalline structure of the HGM@C and PU composites at 2θ = 5°–90° with a 5 °/min scan rate. Fourier transform infrared spectroscopy (FTIR, Nicolet 6700, Thermo Fisher, America) determined the chemical structures of HGM, HGM@C, and PU composites across 4000–450 cm^−1^ with 4 cm^−1^ resolution. X-ray photoelectron spectroscopy (XPS, AXIS UltraDLD, Shimadzu, China) analyzed the element and valence states in HGM@C. Raman spectrometer (Renishaw inVia Qontor, Renishaw, Britain) assessed graphitization in HGM@C over 100–4000 cm^−1^. The thermal properties of HGM and HGM@C were investigated by a Thermogravimetric Analysis (TGA, TGA 550, TA instruments, America) system at a ramp rate of 10 °C/min from room temperature to 600 °C under an air atmosphere. Meanwhile, the thermal properties of PU composites were measured at a ramp rate of 10 °C/min from room temperature to 500 °C under a nitrogen atmosphere, and the reported values represented the typical value of three measurements of each PU composite. The carbon coating of HGM@C was observed by transmission electron microscopy (TEM, JEM-2100F, JEOL, Japan).

Mechanical testing: The universal testing tensile machine (Zwick Roell Z020, ZwickRoell, Germany) performed tensile and compression tests. The parallel size of samples for the tensile test was 16 mm × 4 mm × 2 mm (length × width × thickness) with a 50 mm/min extension rate. The size of the samples for the compression test was 25 mm × 10 mm × 10 mm with a 1 mm/min compression rate. A shore hardness tester (LX-A, Shanghai Siwei Instrument Manufacturing Co., Ltd., Shanghai, China) was used to determine the hardness of samples. Reported mechanical performance represented the typical value of five measurements per sample. Damping properties of different PU elastomers and composites were evaluated on a dynamic analyzer (DMA 850, TA instruments, America) using a tensile clamp. The size of the samples was about 20 mm × 5 mm × 2 mm, and the temperature was swept from −70 to 70 °C at a 3 °C/min heating rate. The reported values represented the typical value of three measurements per sample. All of the specimens were subject to a 0.1% strain amplitude oscillation at 1 Hz frequency.

Acoustic testing: Sound insulation performance was characterized using a standing wave tube (SW230, Beijing Prestige Acoustic Technology Co., Ltd., Beijing, China) with a four-sensor measurement method [39]. Cylindrical PU composite specimens with Φ30 mm × 5 mm and Φ100 mm × 5 mm were prepared to evaluate transmission loss (TL) across the 1000–6300 Hz and 500–1600 Hz frequency range, respectively.

## 3. Results and Discussion

### 3.1. Design of PU with Room Temperature Damping Property

#### 3.1.1. Effect of Molecular Weight of PTMG

**Figure 2:**
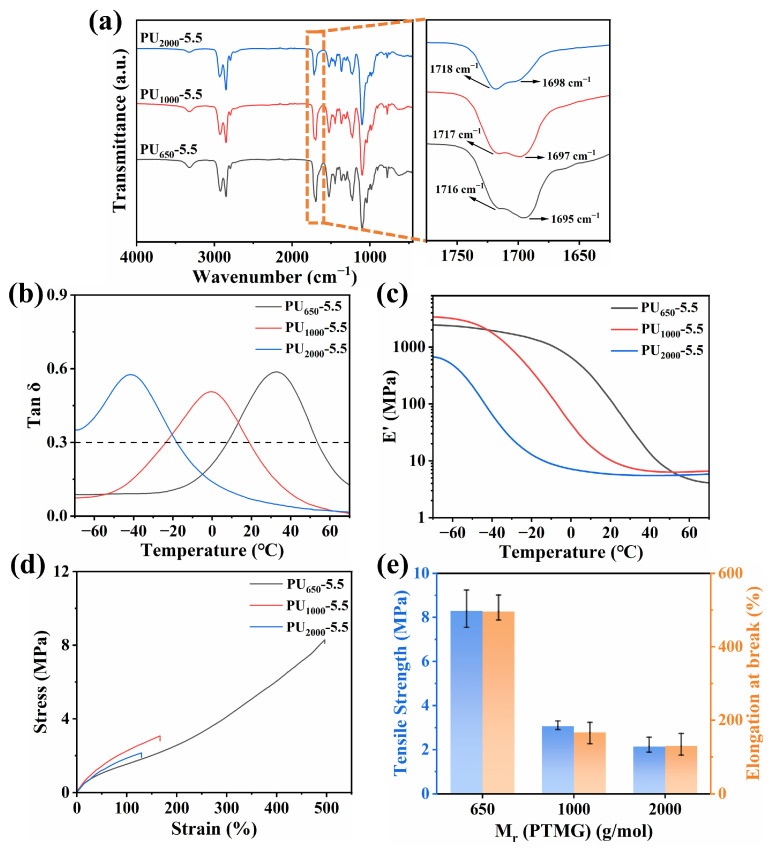
(**a**) FTIR spectrum; (**b**) DMA loss factor (tanδ) curves; (**c**) DMA storage modulus (E’) curves; (**d**) typical stress–strain curves; (**e**) tensile strength (Rm) and elongation at break (At) of PU_2000_-5.5, PU_1000_-5.5, and PU_650_-5.5.

The molecular weight of the soft segment significantly influences the effective damping temperature range of PU. To systematically study this effect, PTMG grades with average molecular weights of 650, 1000, and 2000 g/mol were selected, while maintaining a fixed mole ratio of HMDI:PTMG:GL at 10:5.5:3. Accordingly, the resulting PU samples were designated as PU_2000_-5.5, PU_1000_-5.5, and PU_650_-5.5, reflecting the PTMG molecular weight. FTIR analysis, shown in Figure 2a, revealed no detectable absorption peaks at 2250 cm^−1^ (-NCO), and the vanishing of the wide -OH peak in PTMG and GL, shown in Figure S2, confirmed the near-complete consumption of these functional groups. A relatively sharp absorption peak that appeared near 3300 cm^−1^ was related to the -NH- group of -NHCOO-. Due to the effect of hydrogen bonding, the C=O stretching vibration peak of -NHCOO- was differentiated and appears near 1720 cm^−1^ and 1697 cm^−1^. Increasing the soft segment molecular weight enhanced the free C=O peak intensity at 1720 cm^−1^ while reducing the hydrogen-bonded C=O signal near 1697 cm^−1^ in Figure S1.

As the PTMG molecular weight decreased, the glass transition temperature (T_g_) increased from −41.58 °C (PU_2000_-5.5) to 32.85 °C (PU_650_-5.5) due to elevated crosslinking density restricting chain mobility, and the effective damping temperature range (EDTR) of PU_650_-5.5 was 7.53–53.89 °C. E’ at 20 °C rose substantially from 5.8 MPa (PU_2000_-5.5) to 118.9 MPa (PU_650_-5.5). The increase in crosslinking density effectively improved the storage modulus, reflecting enhanced segmental interactions. Mechanical testing further demonstrated that higher crosslinking density increased R_m_ and A_t_. Moreover, the decrease in molecular weight in the soft segment promoted the formation of hydrogen-bonded domains, and the interaction between molecular segments was enhanced, thus greatly improving mechanical properties. Based on the optimal balance of mechanical strength and damping performance, PU_650_-5.5 with the highest storage modulus was selected for subsequent optimization studies.

#### 3.1.2. Effect of the Mole Ratio of PTMG and GL

**Figure 3:**
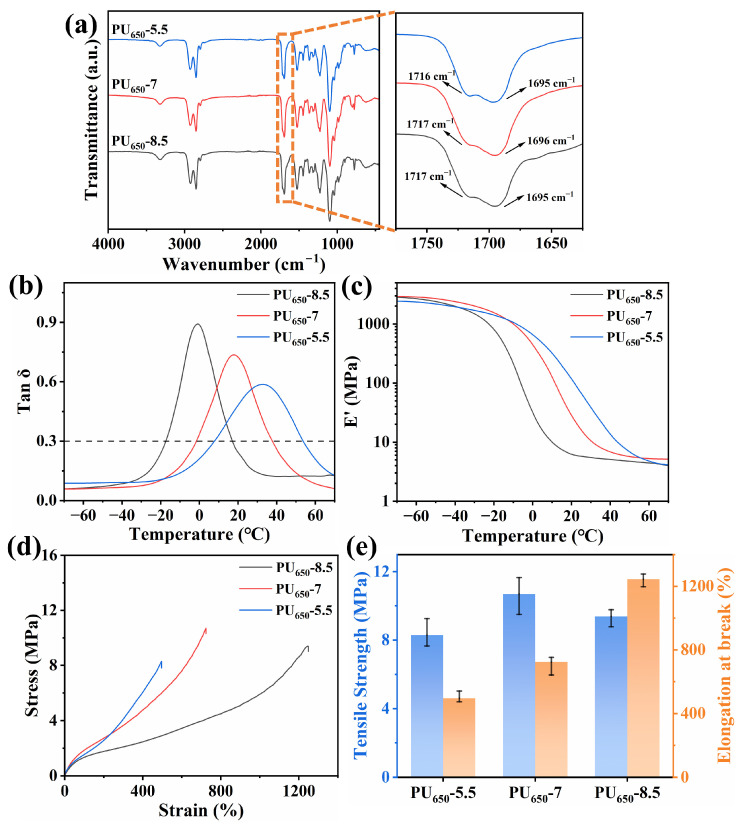
(**a**) FTIR spectrum; (**b**) DMA loss factor (tanδ) curves; (**c**) DMA storage modulus (E’) curves; (**d**) typical stress–strain curves; (**e**) tensile strength and elongation at break of PU_650_-5.5, PU_650_-7, and PU_650_-8.5.

As the EDTR of PU_650_-5.5 is over room temperature and tanδ_max_ is relatively low, the mole ratio of PTMG 650 was increased to optimize the damping property of PU. The two new samples were named as PU_650_-7 and PU_650_-8.5 according to the PTMG 650 molar content. The FTIR spectrum in Figure 3a shows that the characteristic peaks in PU_650_-7 and PU_650_-8.5 were almost the same as PU_650_-5.5. With the increase in PTMG 650 content, T_g_ decreased progressively, shifting the EDTR toward lower temperatures. Tanδ_max_ increased significantly due to reduced steric hindrance of PU molecular chain as HMDI proportion decreased, and the reduction in GL lessened the number of crosslinking points, which enhanced chain mobility and internal friction. Storage modulus (E’) at 20 °C declined from 28.1 MPa (PU_650_-7) to 6.2 MPa (PU_650_-8.5), indicating reduced stiffness.

Mechanical testing further revealed that higher PTMG content simultaneously enhanced both A_t_ and R_m_ in PU_650_-7, with R_m_ rising from 8.28 MPa to 10.7 MPa and A_t_ increasing from 500% to 724%. When the degree of crosslinking was further reduced, excessive inelastic deformation in PU_650_-8.5 occurred (A_t_ = 1248%). However, R_m_ also fell to 9.4 MPa due to the poor stiffness. Critically, PU_650_-7 exhibited an optimal EDTR of −1.68 to 37.78 °C, which aligns with typical ambient conditions while maintaining adequate mechanical integrity.

#### 3.1.3. Effect of the Content of DMTDA

To improve the mechanical properties of PU, chain extender DMTDA with a rigid benzene ring was incorporated while maintaining the isocyanic acid index of 1. Three new samples were synthesized based on the mole ratio of GL and DMTDA, designated as PU_650_-7(6/9), PU_650_-7(2/9), and PU_650_-7(0/9). FTIR analysis confirmed complete reaction of -OH and -NCO groups, and the double peak of -NH_2_ in 3300–3500 cm^−1^ (Figure S2) vanished, revealing that -NH_2_ in DMTDA almost reacted. Due to the addition of DMTDA, reaction between -NH_2_ and -NCO formed -NHCONH- linkages, and ordered hydrogen bonding of C=O formed between molecular chains, manifested by a new peak at 1632 cm^−1^ in the FTIR spectrum [40,41]. The hydrogen bonding content in PU was quantified through peak deconvolution of the FTIR absorption band from 1600 to 1760 cm^−1^, as shown in Figure S3. Table 2 confirmed that the total degree of hydrogen-bonded C=O groups increased with the enhancement of DMTDA content.

**Figure 4:**
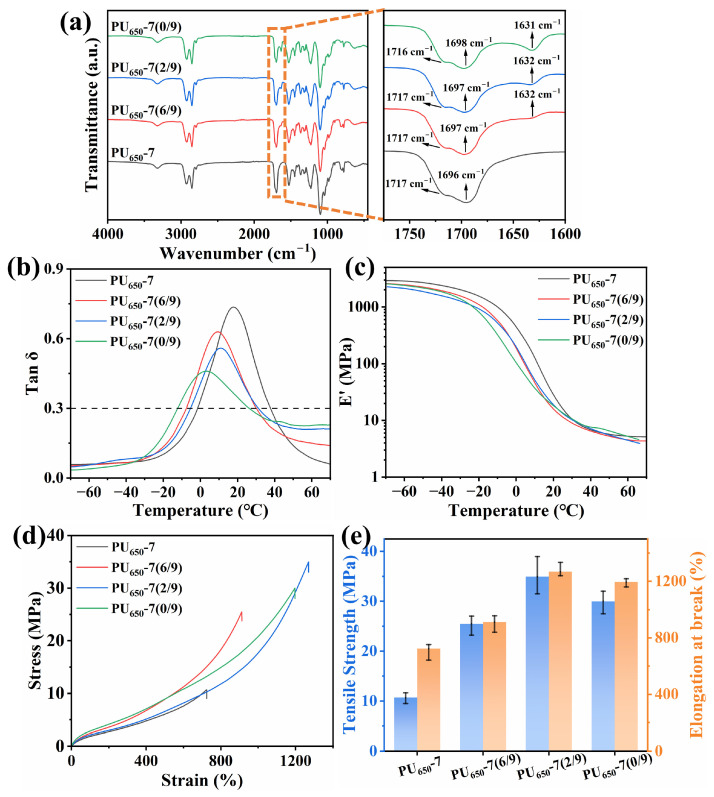
(**a**) FTIR spectrum; (**b**) DMA loss factor (tanδ) curves; (**c**) DMA storage modulus (E’) curves; (**d**) stress–strain curves; (**e**) tensile strength and elongation at break of PU_650_-7, PU_650_-7(6/9), PU_650_-7(2/9), and PU_650_-7(0/9).

With increasing DMTDA content, T_g_ shifted to lower temperatures. This trend is attributed to the reduction in crosslinking points generated by GL, enhancing the mobility of PU molecular chain segments. Meanwhile, the EDTR of PU also gradually shifted to lower temperatures. The benzene ring in DMTDA increased steric hindrance within PU segments, while ordered hydrogen bonding further restricted chain segment movement, leading to a gradual decrease in tanδ_max_. However, the combined effects of the rigid benzene ring in DMTDA and enhanced hydrogen bonding failed to compensate for the stiffness loss caused by reduced crosslinking. Consequently, E’ at 20 °C slightly decreased compared to PU_650_-7. Figure 4d shows that both R_m_ and A_t_ improved simultaneously, resulting from longer molecular chains in the crosslinked network and enhanced hydrogen bond interactions. Specifically, PU_650_-7(2/9) achieved an A_t_ of 1269% with an R_m_ of 35.0 MPa. In contrast, PU_650_-7(0/9) without GL addition exhibited reduced A_t_ (1197%) due to insufficient crosslinking points for network formation. Damping properties of PU materials fabricated in this section are listed in Table 3. The *TA* value could be calculated as the total area within the EDTR:

$$TA=\int_{T_{1}}^{T_{2}}\tan\delta dT$$

where *tanδ* is the loss factor, and *T*_1_ and *T*_2_ are initial and final temperatures of EDTR, respectively [42]. Based on the integrated damping and mechanical properties as shown in Figure S4, PU_650_-7(6/9) with balanced performance (tanδ_max_ = 0.630, EDTR: −7.32–30.19 °C, R_m_ = 25.5 MPa, A_t_ = 912%, E’ = 15.7 MPa at 20 °C) was selected for subsequent studies.

### 3.2. Characterization of HGM@C

**Figure 5:**
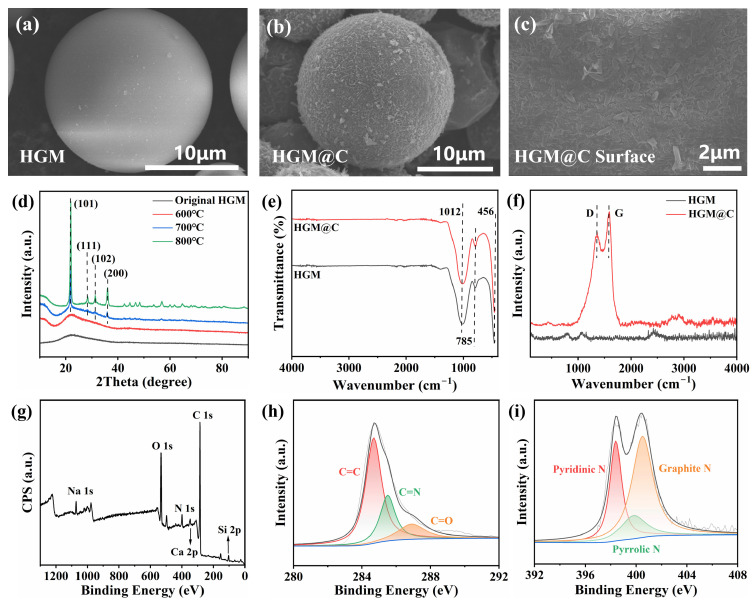
SEM images of (**a**) original HGMs; (**b**) carbon coated HGMs; (**c**) the surface of HGM@C; (**d**) XRD patterns of HGMs under different heat treatment temperatures; (**e**) FTIR spectrum; (**f**) Raman spectrum; (**g**) XPS spectra of HGM@C; high-resolution XPS spectra of (**h**) C 1s; (**i**) N 1s of HGM@C.

Figure 5a reveals that the pristine hollow glass microspheres (HGMs) exhibited a relatively smooth surface. After PDA coating and subsequent carbonization, carbon nanosheets formed on the HGM surface, increasing surface roughness and enhancing surface area. The TEM image in Figure S5 also clearly reveals the presence of carbon coating on the HGM surface. Notably, the coating thickness is non-uniform at the microscopic level. This observation aligns with the rough texture morphologies of HGM@C depicted in Figure 5b,c.

Notably, the spherical morphology of the HGMs was preserved after carbonization at 600 °C. Given that HGMs consist of sodium and calcium silicates, high-temperature heat treatment would induce crystallization, leading to embrittlement and structural fracture of the HGMs. As demonstrated in Figure 5d, the HGMs maintained their amorphous state after 600 °C heat treatment, consistent with the original material. However, at elevated calcination temperatures (700 °C and 800 °C), distinct diffraction peaks emerged in the XRD pattern. A prominent peak at about 2θ ≈ 21.7° corresponding to the (110) crystallographic plane of cristobalite, and some additional peaks occurred at other angles, confirming crystallization of the HGMs. To ensure retention of spherical morphology, 600 °C was selected as the carbonization temperature.

Figure 5e indicates minimal changes in the FTIR spectra of HGMs and HGM@C. Since the original HGMs were not chemically treated, no functional groups such as -OH or -NH_2_ appeared in the 3650–3100 cm^−1^ region. The prominent peak at 1012 cm^−1^ could be regarded as the superposition of the Si-O-Si symmetric stretching vibration peak at 1100–1080 cm^−1^ and the hydrogen-bonded Si-OH vibration peak on the HGM surface at about 960 cm^−1^. In addition, characteristic bending vibration peaks of Si-O-Si and Si-O also appeared at 785 cm^−1^ and 456 cm^−1^, respectively [28].

To further prove the presence of carbon coating, Raman spectroscopy was performed on both HGM and HGM@C. Figure 5f reveals two prominent peaks at 1357 cm^−1^ and 1590 cm^−1^ in HGM@C, corresponding to the typical D-peak and G-peak of carbon materials [43], representing the amorphous and graphitic structures of carbon, respectively. These two peaks were absent in bare HGMs. To further analyze changes between HGM@PDA and HGM@C, peak deconvolution analysis was conducted on Raman spectra within 1000–1800 cm^−1^, as shown in Figure S6. The A band was caused by amorphous carbon and organic functional groups, and the D” band originated from the stretching vibrations of C-C or C=C bonds in the olefin structure [44]. After carbonization at 600 °C, the intensity ratios I_D_/I_G_, I_A_/I_G,_ and I_D”_/I_G_ decreased significantly. The calculated I_D_/I_G_ of HGM@PDA and HGM@C by using the fitting peak areas of the curves were 0.865 and 0.367, respectively, indicating the PDA coating transformed into carbon. As PDA contains -OH and -NH_2_, the strength of the A band was high in HGM@PDA. After heat treatment, PDA converted to an N/O doped carbon layer, enhancing the G-band intensity. As the olefin structure of PDA was preserved during carbonization, the D”-band intensity remained stable. These results collectively validate the successful carbonization of the PDA coating on HGM surfaces.

To determine the elemental composition and valence states of HGM@C, XPS analysis was performed. The spectrum of HGM@C reveals the presence of C, N, O, Si, Na, and Ca elements, consistent with the silicate components (Na/Ca) inherent in HGMs. The high-resolution C1s spectrum was deconvoluted into three characteristic peaks at 284.7 eV, 285.5 eV, and 286.9 eV in Figure 5h, assigned to the C=C, C-N, and C=O bonds, respectively [45]. The dominant C=C sp^2^ hybridization component (60.41% area fraction) represents the typical carbon bonding configuration in carbonaceous materials. The coexistence of C-N and C=O bonds confirms N and O doping within the carbon framework, originating from the carbonization of PDA. Similarly, the N 1s spectrum was fitted with three peaks at 398.4 eV (pyridinic N), 399.8 eV (pyrrolic N), and 400.5 eV (graphitic N), respectively [46]. Collectively, these XPS results verify the successful coating and carbonization of PDA on the HGM surface.

To further assess the amount of deposited carbon, thermogravimetric analysis of HGM and HGM@C was performed under an air atmosphere. Figure S7 shows that for HGMs, the weight loss ratio reached 98.90% at 600 °C and stabilized above 700 °C at 98.75%. For HGM@C, the weight loss ratio stabilized above 700 °C at 92.06%. Given that HGM@C underwent previous heat treatment at 600 °C, we attribute the minimal weight loss observed above 600 °C in the HGM@C (about 0.15%) primarily to the HGM component. Consequently, the significant weight loss difference of 7.79% is attributed to the oxidation of the carbon coating.

### 3.3. Characterization of HGM@C/PU

**Figure 6:**
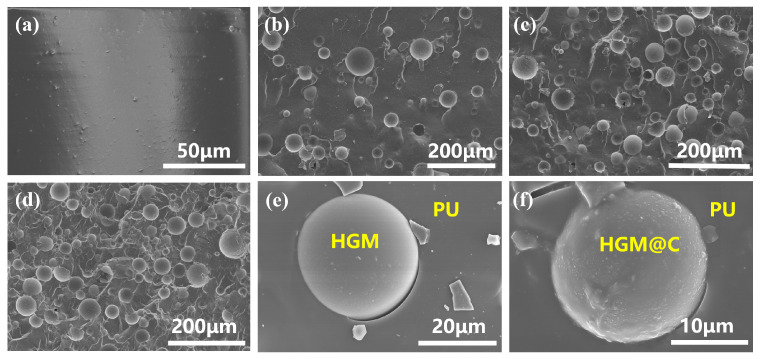
(**a**–**d**) Fracture surface of pure PU, PU-5C, PU-10C, and PU-15C in liquid nitrogen; (**e**,**f**) fillers debonding in tensile fracture surface of PU-10 and PU-10C composites.

In Figure 6a–d, pure PU exhibited a relatively smooth fracture surface, while PU-5C and PU-10C composites showed uniform dispersion of HGM@C within the PU matrix without flotation. PU-15C displayed HGM@C agglomeration due to enhanced prepolymer viscosity impeding particle dispersion, which compromises mechanical properties [47]. EDS elemental mapping of Si (Figure S8) confirmed that the spherical features observed in Figure 6e,f correspond to the debonding fillers within the PU matrix. Bare HGMs maintained smooth surfaces after extraction, whereas HGM@C developed roughened textures with distinct protrusions. This contrasts with the pristine nanosheet carbon coating observed in Figure 5c, suggesting PU matrix adhesion to HGM@C during tensile failure.

The molecular structures of PU composites were characterized by FTIR, revealing the characteristic peak at 3323 cm^−1^ assigned to N-H stretching vibrations. Almost no -NCO stretching peak was observed at 2250 cm^−1^, confirming complete -NCO reaction. Peaks at 1038 cm^−1^ and 901 cm^−1^ represented the double ring structure in the HMDI monomer. Hydrogen-bonded C=O stretching vibrations appeared between 1600 and 1750 cm^−1^, consistent with what is described in 3.1.3. Enhanced Si-O bond intensity at about 460 cm^−1^ with increasing HGM and HGM@C content confirmed successful filler incorporation. No significant changes in the PU matrix molecular structure were induced by filler addition. In the XRD spectrum, a broad peak appeared at 2θ ≈ 19.5°, revealing the amorphous state of pristine PU. The reduced intensity of this peak with increasing HGM and HGM@C content indicated that rigid fillers restrict molecular chain crystallization.

**Figure 7:**
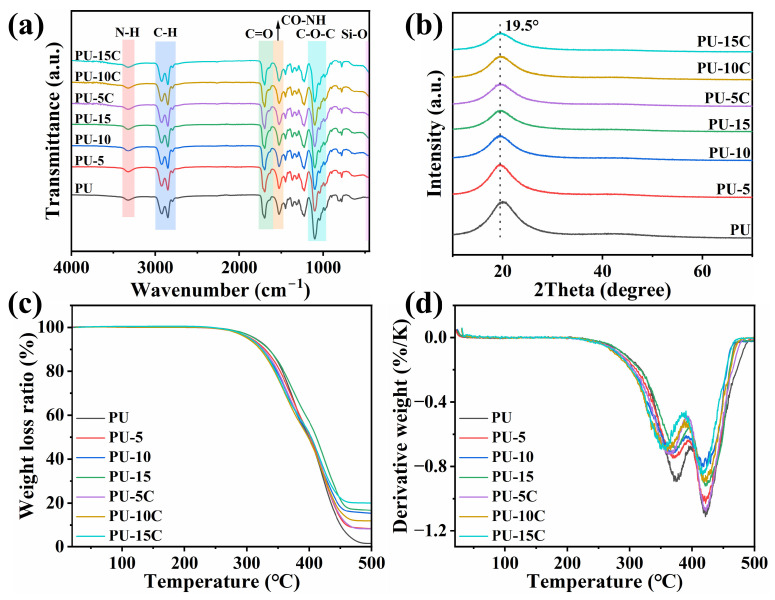
(**a**) FTIR spectrum; (**b**) XRD spectrum; (**c**,**d**) TG and DTG curves of PU composites.

The derivative thermogravimetry (DTG) curves in Figure 7d revealed a two-stage thermal decomposition process for PU. The first stage in 260–397 °C is attributed to weight loss primarily from the hard segment, while the soft segment decomposition dominated the second step in 397–470 °C. Pure PU exhibited minimal residual mass (1.56 wt% at 500 °C), indicating near-complete decomposition. Incorporation of HGMs and HGM@C increased the residual weight due to the thermal stability of rigid inorganic fillers at 500 °C. Minor variations in residual mass between PU-10/PU-10C and PU-15/PU-15C composites suggested localized inhomogeneity without altering the overall trend. The weight loss rate decreased with increasing filler loading. The initial decomposition temperatures (95% of the remaining weight) of PU composites were almost the same, and thermal stability above 400 °C was improved due to the barrier effect of rigid HGMs, while the carbon coating improved heat distribution.

**Figure 8:**
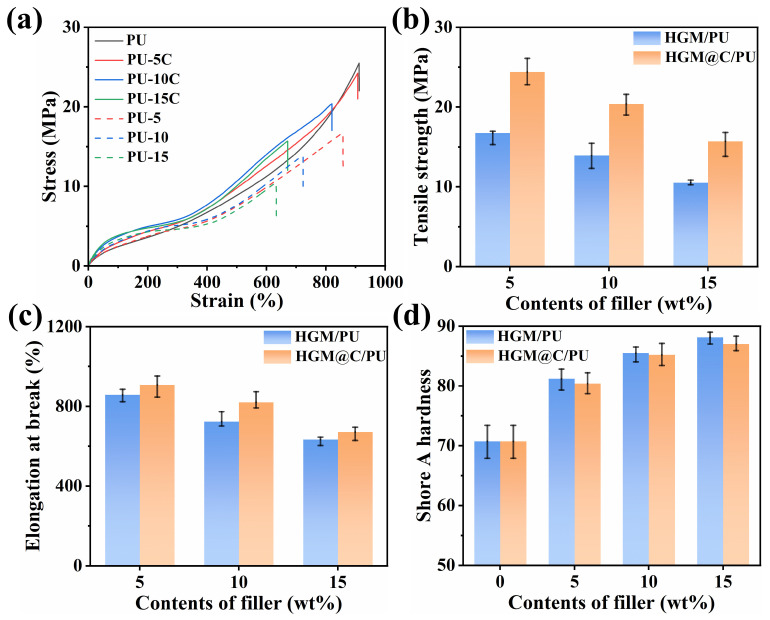
(**a**) Tensile stress–strain curves; (**b**) tensile strength; (**c**) elongation at break; (**d**) Shore A hardness of PU composites.

In Figure 8a, the stress–strain curves of PU composites before 300% strain were very close. At higher deformations (≥600%), the molecular chain became oriented along the tensile direction, increasing the slope of stress–strain curves. The rigid HGM and HGM@C hindered PU chain mobility and extension, reducing orientation-induced reinforcement at equivalent strains and thereby synchronously decreasing R_m_ and At. The R_m_ of the PU matrix was 25.5 MPa, while the R_m_ values of PU-5 and PU-5C were 16.7 MPa and 24.4 MPa, respectively. In PU-15 and PU-15C composites, filler aggregation altered the failure mechanism; the fracture mode transitioned from ductile failure of the matrix to brittle filler fracture of the filler particles, inducing void formation during tensile deformation and thereby deteriorating mechanical properties. HGM@C exhibited enhanced interfacial interactions with the PU matrix due to surface roughening, as mentioned in Figure 5b,c. The increased interfacial area dissipated more fracture energy, significantly improving R_m_ and A_t_ in HGM@C/PU composites versus uncoated HGM/PU at identical filler loading [48] in Figure 8b,c. After carbon coating treatment, the R_m_ of PU composites with the same filler proportions (5, 10, and 15 wt%) increased by 46.1%, 46.8%, and 49.5%, respectively.

Shore A hardness was performed to further evaluate the stiffness of PU composites. Figure 8d demonstrates that composite hardness increased with higher filler loading. No significant difference in hardness was observed between HGM/PU and HGM@C/PU systems at identical filler mass fractions. However, the carbon coating occupies partial volume within the PU matrix, marginally reducing the effective volume fraction of hollow-structured HGM in HGM@C. Consequently, HGM@C/PU composites exhibited slightly lower hardness than HGM/PU at equivalent weight ratios.

**Figure 9:**
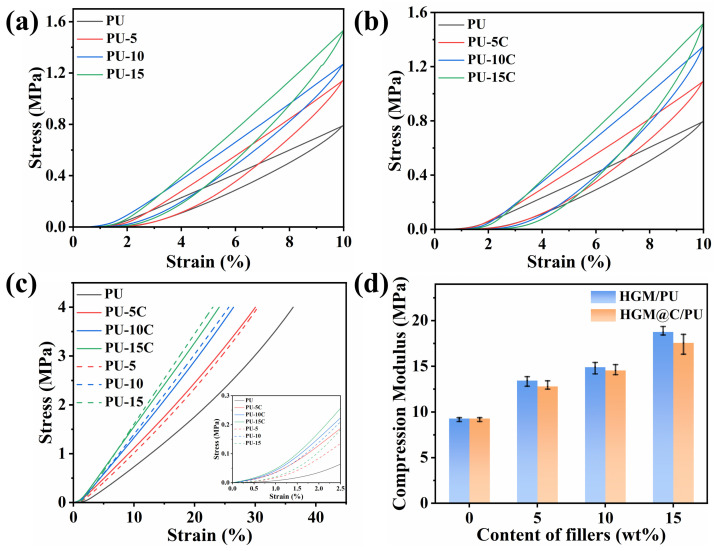
Compressed hysteresis loop stress–strain curves of (**a**) HGM/PU; (**b**) HGM@C/PU composites with strain of 10%; (**c**) compressed stress–strain curves of PU composites; (**d**) compression modulus of HGM/PU and HGM@C/PU composites.

The hysteresis loop area in compression stress–strain curves in Figure 9a,b represents energy dissipation in PU composites. As reported, the damping properties comprise the intrinsic damping from molecular chain mobility and the interface damping from filler-matrix friction [49]. Enhanced composite stiffness and restricted chain motion due to rigid HGM reduced intrinsic damping. The energy density (*h*) of the cyclic stress–strain curve represents the dissipative energy under cyclic deformation, while the strain energy density (*w*) indicates the undissipated energy under cyclic deformation. The dissipation coefficient (*DE*) is defined as the ability of the material to dissipate energy [50,51], which can be calculated as:

$$DE=\frac{h}{h+w}\times 100\%$$

As shown in Table 4, the calculated *DE* value of HGM@C/PU increased from 23.95% to 35.13% with higher filler loading, while HGM/PU exhibited minimal *DE* variation, confirming superior interfacial friction and damping capacity at carbon-coated interfaces. To evaluate the compressive properties of PU composites, compression tests at a maximum stress of 4 MPa were conducted. The deformation of pure PU under 4 MPa was 36.25%, and the addition of HGMs effectively improved compressive performance. The strain of PU-5, PU-10, and PU-15 under 4 MPa was 30.44%, 25.64%, and 23.00%, respectively, while the strain of PU-5C, PU-10C, and PU-15C under 4 MPa was 30.03%, 26.42%, and 24.06%, respectively. HGM@C/PU composites showed marginally higher deformation than HGM/PU, which may be attributed to reduced effective hollow volume fraction as carbon coating occupied a little space and the slight crystallinity changes of HGM@C during heat treatment compared with untreated HGMs. Consequently, HGM@C/PU exhibited a lower compression modulus than HGM/PU at equivalent filler content, as shown in Figure 9d. For viscoelastic damping materials subjected to low-strain conditions, the stress–strain relationship exhibits significant nonlinearity (Figure 9c). Therefore, the compressive modulus was determined from the region at strains of 5–15%, where material behavior becomes predominantly linear. Crucially, both pressure resistance and compression modulus reductions remained limited, demonstrating preserved hollow structure functionality and compressive performance after carbon coating.

**Figure 10:**
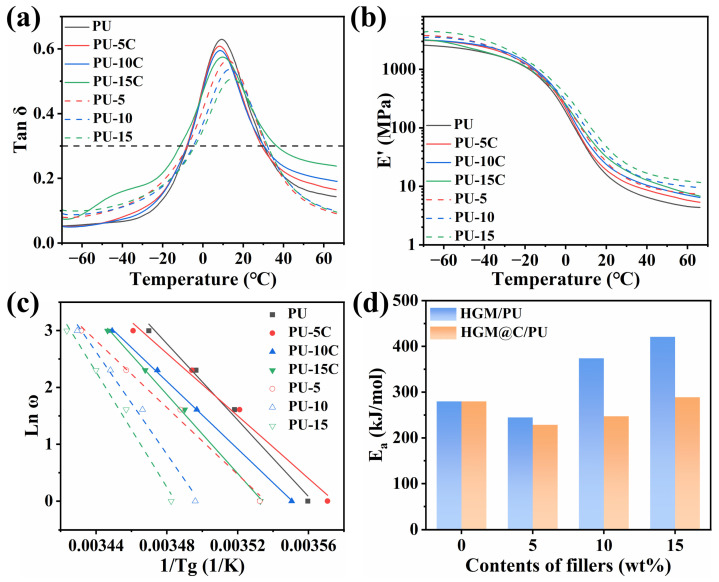
(**a**) Loss factor-temperature curves; (**b**) storage modulus-temperature curves; (**c**) DMA extrapolation curve, and (**d**) segment activation energy of PU composites derived from the Muller and Huff equations.

DMA results indicate comparable EDTR of PU-5C and PU-10C with the PU matrix. Enhanced composite rigidity restricted molecular chain segment mobility, leading to reduced tanδ_max_ versus pure PU. However, HGM@C/PU exhibited higher tanδ_max_ than HGM/PU at equivalent filler loading, as compared in Figure 10a and Table 5. This enhancement arose from improved interfacial compatibility via the carbon interlayer. The energy generated by the interfacial friction between PU molecular chain movement and HGM@C was more easily dissipated by the carbon layer with better thermal conductivity. In addition, the increased interface area between the PU matrix and filler provided the contact probability between dispersed HGM@C, promoting energy dissipation under vibrational loading. In PU-15C, reduced bulk density and increased carbon mass fraction facilitated more continuous thermal conductivity networks. Consequently, while tanδ_max_ decreased, EDTR of PU-15C broadened. Table 5 confirms significantly increased TA values for HGM@C/PU versus HGM/PU composites.

Based on the DMA curves of PU composites conducted at frequencies of 1, 5, 10, and 20 Hz, and combined with the Arrhenius equation and Muller–Huff equation [10], the segment activation energy *E_a_* was calculated according to the following formula:

$$E_{a}=R\frac{dln\omega }{d(1/T)}$$

where *R* is the gas constant, *ω* is the frequency, and *T* is the absolute temperature. The fitting results are shown in Figure 10c,d. *E_a_* of pure PU was 279.7 kJ/mol. At low filler content in PU-5 and PU-5C, *E_a_* decreased because of the additional internal friction between the PU segment and fillers. However, when the content of fillers increased to 10 wt%, *E_a_* of PU-10 greatly increased to 373.6 kJ/mol, while PU-10C showed a moderate increase to 247.2 kJ/mol. At 15 wt% filler loading, *E_a_* of PU-15 reached 420.9 kJ/mol, while *E_a_* of PU-15C maintained a relatively low value of 288.8 kJ/mol. Although a large volume of HGM@C increased the rigidity of composites and movement of the segment was hindered, the carbon coating decreased the difficulty of chain segment movement. This phenomenon could be attributed to the interfacial damping effects of HGM@C. The carbon nanosheets coating on the HGM surface disrupts the ordered arrangement of PU soft segments in proximity to HGM@C. Such disruption promotes molecular rearrangement, particularly under high-frequency conditions, thereby enhancing energy dissipation through friction between molecular chains and between chains and the HGM@C interface. The interface friction was larger than HGM/PU because of the rougher surface of HGM@C, so more heat was generated because of the slip between HGM@C and PU molecules. Compared to thermally insulated HGMs, the carbon coating could help conduct the heat, accelerating the movement of the PU molecular chain around HGM@C.

**Figure 11:**
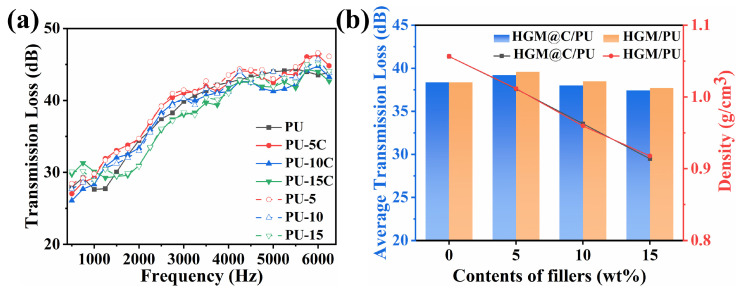
(**a**) Sound insulation curves of PU composites; (**b**) average transmission loss and density of PU composites.

Thanks to the hollow structure of HGMs, composites with HGMs can overcome the mass law limitations for developing lightweight sound insulation materials. To verify carbon coating effects, the sound insulation properties of HGM@C/PU were evaluated. The transmission loss (TL) of PU composites in 500–6300 Hz is shown in Figure 11a.

Despite density reduction as filler loading increased (volume density of pure PU is 1.057 g/cm^3^, while the volume densities of PU-15 and PU-15C are 0.914 and 0.918 g/cm^3^, respectively, decreasing by nearly 13.2%), TL value over 2000 Hz showed no significant decrease, and average transmission loss (ATL) across 500–6300 Hz still maintained over 37 dB. The average transmission loss (ATL) of PU-5 and PU-5C was 39.59 dB and 39.21 dB, respectively, which were higher than pure PU with 38.38 dB. This could be explained by the confined air in a narrow space and the dampening effect of sound waves in the cavities of HGMs [23,39]. With further decreases in volume density, the ATL values of PU-10 and PU-10C reduced to 38.49 and 38.00 dB, respectively, and the ATL values of PU-15 and PU-15C reduced to 37.72 and 37.43 dB, respectively. This could be attributed to mass law. Lower density would lead to a decrease in the sound insulation effect. In Figure 11b, the densities of HGM/PU and HGM@C/PU with the same filler content were almost the same, and the ATL values exhibited a small difference of 0.3–0.5 dB, which could be attributed to the effect that carbon coating occupied a small amount of space and weakened the damping effect of sound waves in HGMs. Overall, the hollow structure of the HGMs was preserved during the carbon coating procedure, and the sound insulation property of the PU composites was maintained.

## 4. Conclusions

A casting PU with balanced damping and mechanical properties was successfully designed by optimizing monomer types and ratios, exhibiting a tensile strength of 25.5 MPa and an effective damping temperature range from −7.32 to 30.19 °C. Meanwhile, HGM@C was synthesized through in situ polymerization of polydopamine on HGMs, followed by carbonization, with successful carbon coating confirmed through various characterization techniques. The HGM@C fillers demonstrate uniform dispersion within the PU matrix, significantly enhancing composite hardness and compressive deformation resistance. Tensile strength of HGM@C/PU substantially exceeds that of HGM/PU due to superior interfacial bonding between the carbon-coated fillers and PU matrix. Although high filler loading inevitably reduces the inherent damping capacity of PU, the carbon coating mitigates this decline in damping loss performance. For equivalent filler ratios, HGM@C/PU exhibits higher TA values and activation energy than HGM/PU, attributable to enhanced interfacial damping loss. Critically, HGM@C/PU maintains equivalent acoustic insulation performance to HGM/PU, as carbon coating preserves the hollow structure of HGMs. This confirms the applicability of HGM@C/PU in lightweight damping and sound-insulating composites used for vibration-damping/isolation gaskets in rail transit systems, lightweight buffer components in aircraft, and acoustic skins on underwater vehicles.

## Figures and Tables

**Table 1:** The monomer component of PU elastomers.

Sample	HMDI (mmol)	PTMG (mmol)	DMTDA (mmol)	GL (mmol)
PU_2000_-5.5	10	5.5 (M_r_ = 2000 g/mol)	0	3
PU_1000_-5.5	10	5.5 (M_r_ = 1000 g/mol)	0	3
PU_650_-5.5	10	5.5 (M_r_ = 650 g/mol)	0	3
PU_650_-7	10	7 (M_r_ = 650 g/mol)	0	2
PU_650_-8.5	10	8.5 (M_r_ = 650 g/mol)	0	1
PU_650_-7(6/9)	10	7 (M_r_ = 650 g/mol)	1.5	1
PU_650_-7(2/9)	10	7 (M_r_ = 650 g/mol)	2.25	0.5
PU_650_-7(0/9)	10	7 (M_r_ = 650 g/mol)	3	0

**Table 2:** Summary of the ratios of C=O peak areas in different environments.

Assignment		Wavenumber (cm^−1^)				Area (%)			
		PU_650_-7	PU_650_-7(6/9)	PU_650_-7(2/9)	PU_650_-7(0/9)	PU_650_-7	PU_650_-7(6/9)	PU_650_-7(2/9)	PU_650_-7(0/9)
-NHCOO-	Free	I (1721)	I (1720)	I (1720)	I (1720)	18.2	8.2	9.8	9.5
	H-bonded (ordered)	II (1697)	II (1703)	II (1701)	II (1701)	81.8	60.3	62.4	57.4
-NHCONH-	Free	III /	III (1692)	III (1692)	III (1693)	/	9.4	4.3	3.5
	H-bonded (disordered)	IV /	IV (1665)	IV (1658)	IV (1653)	/	13.4	18.5	21.4
	H-bonded (ordered)	V /	V (1636)	V (1633)	V (1631)	/	8.7	5.0	8.2
Total degree of H-bonded						81.8	82.4	84.2	86.2

**Table 3:** Damping property of PU materials.

	Peak Position		Temperature Range of Effective Damping (°C)		E’ at 20 °C (MPa)	*TA* Value
	T_g_ (°C)	tanδ_max_	(T1, T2)	ΔT		
PU_2000_-5.5	−41.58	0.576	(−70, −18.20)	51.8	5.8	25.721
PU_1000_-5.5	−0.88	0.507	(−23.67, 19.01)	42.68	10.2	18.350
PU_650_-5.5	32.85	0.587	(7.53, 53.89)	46.36	118.9	23.407
PU_650_-7	17.69	0.738	(−1.68, 37.78)	39.46	28.1	22.033
PU_650_-8.5	−0.61	0.894	(−17.52, 17.18)	34.70	6.2	21.345
PU_650_-7(6/9)	8.36	0.630	(−7.32, 30.19)	37.51	15.7	18.763
PU_650_-7(2/9)	10.77	0.559	(−5.50, 31.38)	36.88	18.9	16.385
PU_650_-7(0/9)	3.38	0.460	(−12.41, 26.42)	38.83	18.3	15.708

**Table 4:** Compression energy dissipation abilities of PU composites.

	*h*	*w*	*DE* (%)
PU	0.80	2.54	23.95
PU-5C	1.23	3.23	27.90
PU-10C	1.66	3.37	30.66
PU-15C	2.09	3.62	35.13
PU-5	1.14	3.38	25.38
PU-10	1.16	5.38	21.56
PU-15	1.45	4.73	23.46

**Table 5:** Damping property of PU composites.

Sample	Peak Position		Temperature Range of Effective Damping (°C)		*TA* Value	*E_a_* (kJ/mol)	R^2^ Value
	T_g_ (°C)	tanδ_max_	(T1, T2)	ΔT			
PU	8.36	0.630	(−7.32, 30.19)	37.51	18.763	279.7	0.98644
PU-5C	8.25	0.608	(−8.10, 29.38)	37.48	17.823	228.6	0.98312
PU-10C	8.50	0.595	(−8.11, 30.53)	38.64	17.817	247.2	0.99929
PU-15C	9.92	0.574	(−11.31, 36.35)	47.66	21.260	288.8	0.99679
PU-5	11.83	0.544	(−6.70, 30.62)	37.32	14.955	244.6	0.98437
PU-10	13.33	0.536	(−4.52, 31.88)	36.40	14.741	373.6	0.98433
PU-15	14.45	0.508	(−3.67, 32.31)	35.98	14.152	420.9	0.97933

## Data Availability

The data that support the findings of this study are available on request from the corresponding author.

## Supplementary Materials

The following supporting information can be downloaded at: https://www.mdpi.com/article/10.3390/polym17152115/s1, Figure S1: FTIR spectra and fitting of the C=O stretching vibration peaks within a region of 1670-1750 cm^−1^; (d) hydrogen bonding amount of PU_650_–5.5, PU_1000_–5.5 and PU_2000_–5.5; Figure S2: FTIR spectrum of PU monomers; Figure S3: FTIR spectra and fitting of the C=O stretching vibration peaks within a region of 1760-1600 cm^−1^ for (a) PU_650_-7; (b) PU_650_-7(6/9); (c) PU_650_-7(2/9); (d) PU_650_-7(0/9); Figure S4: Radar plot of various PU matrix; Figure S5: TEM image of HGM@C; Figure S6: Raman peaks of D, G, A, and D” bands of (c) HGM@PDA; (d) HGM@C; Figure S7: TG curves of HGM and HGM@C; Figure S8: Distribution of elements C, O, Si in debonding between PU and (a) HGM; (b) HGM@C.
