# Supplementary material for: Synchronous Improvement of Mechanical and Room-Temperature Damping Performance in Light-Weight Polyurethane Composites by a Simple Carbon-Coating Strategy
Source: Polymers (Basel). 2025 Jul 31;17(15):2115. doi: 10.3390/polym17152115 (PMC12349370; doi:10.3390/polym17152115)
Supplement: Supplementary file 1 [file polymers-17-02115-s001.zip › polymers-3735157-supplementary.pdf]

polymers

MDPI

Supporting Information

Synchronous Improvement of Mechanical and Room-Temperature Damping Performance in Light-Weight Polyurethane Composites by a Simple Carbon-Coating Strategy

Qitan Zheng [1], Zhongzheng Zhu [1], Junyi Yao [1], Qinyu Sun [1], Qunfu Fan [1], Hezhou Liu [1], Qiuxia Dong [2,*] and Hua Li [1,3,*]

[1] State Key Laboratory of Metal Matrix Composites, School of Materials Science and Engineering, Shanghai Jiao Tong University, Shanghai 200240, China; zhengqitan@sjtu.edu.cn (Q.Z.); zzzjtu@sjtu.edu.cn (Z.Z.); yjy2000@sjtu.edu.cn (J.Y.); sunnyqy@sjtu.edu.cn (Q.S.); fanqunfu@sjtu.edu.cn (Q.F.); hzhliu@sjtu.edu.cn (H.L.)

[2] School of Environmental Science and Engineering, Shanghai Jiao Tong University, Shanghai, 200240, China

[3] Inner Mongolia Research Institute, Shanghai Jiao Tong University, Hohhot 010010, China

* Correspondence: dqiuxia2010@sjtu.edu.cn (Q.D.); lih@sjtu.edu.cn (H.L.)

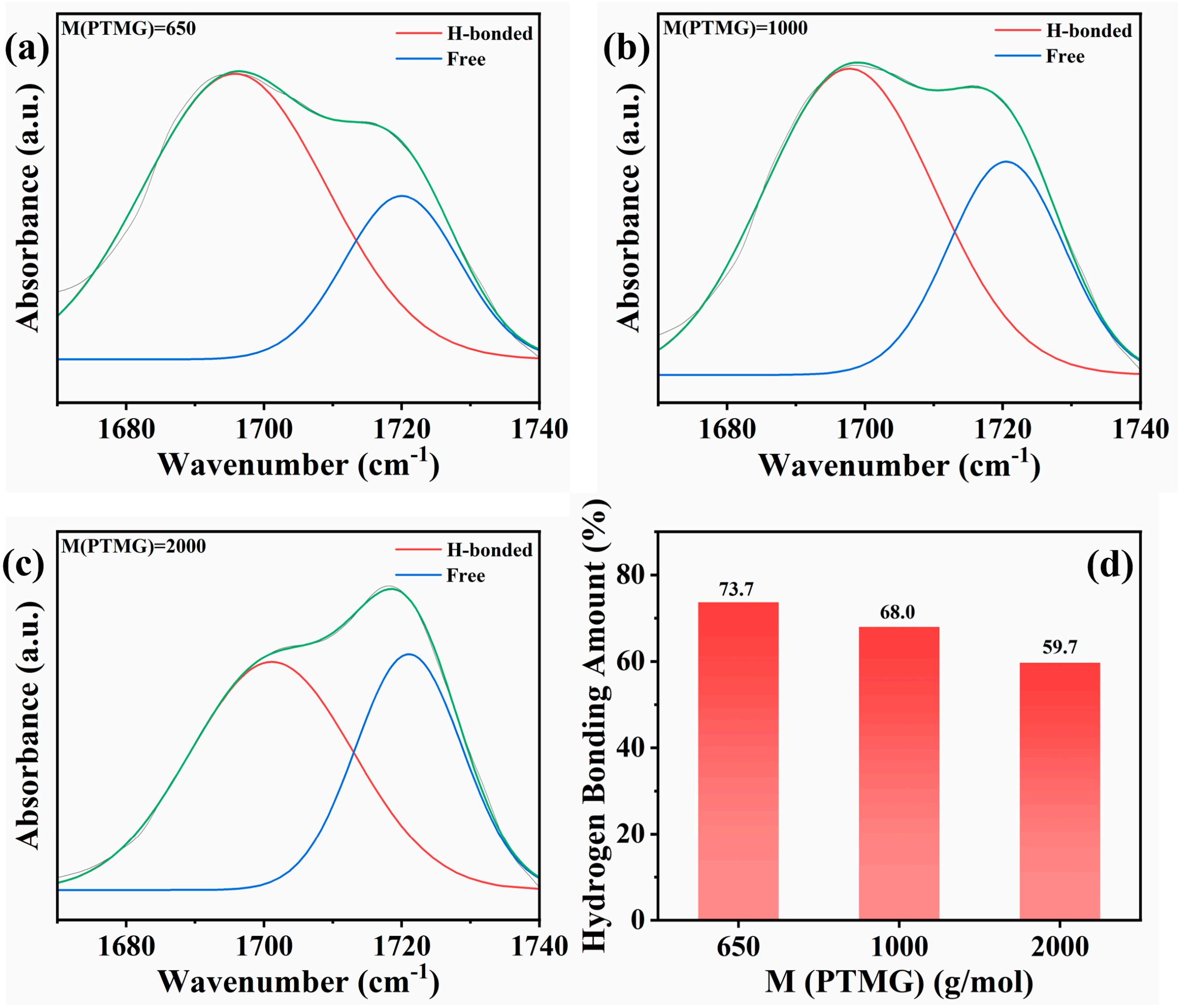

Figure S1 FTIR spectra and fitting of the C=O stretching vibration peaks within a region of 1670–1750 cm^{-1}; (d) hydrogen bonding amount of PU_{650}-5.5, PU_{1000}-5.5 and PU_{2000}-5.5

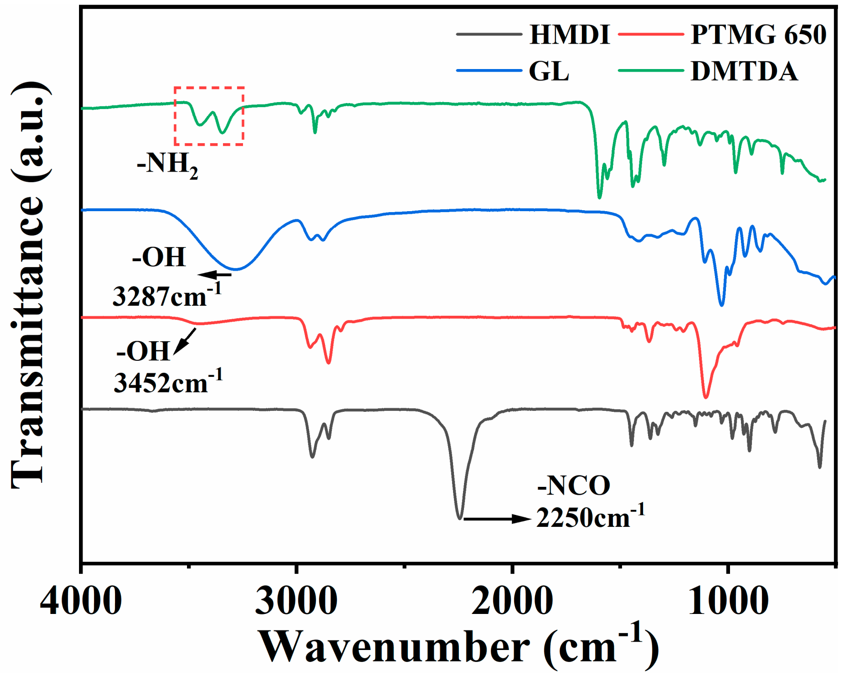

Figure S2 FTIR spectrum of PU monomers

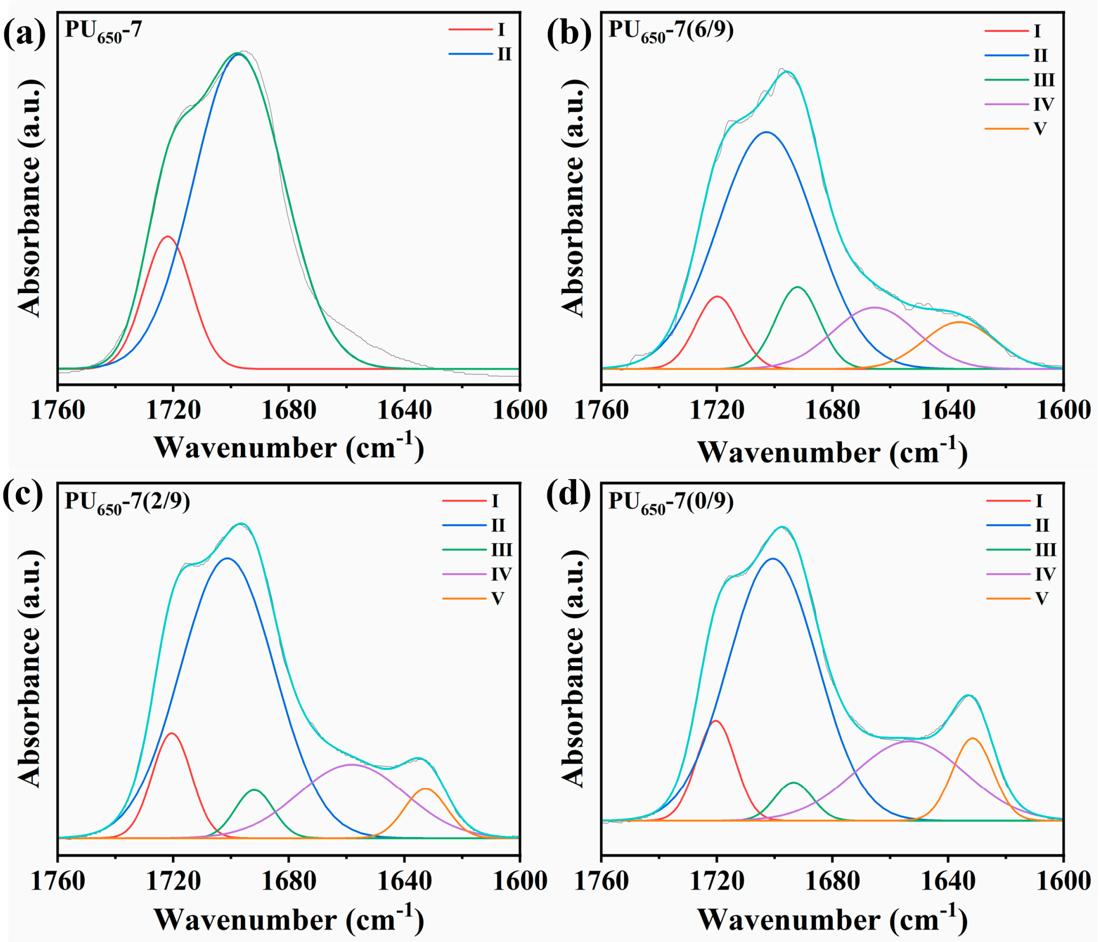

Figure S3 FTIR spectra and fitting of the C=O stretching vibration peaks within a region of 1760–1600 cm^{-1} for (a) PU_{650}-7; (b) PU_{650}-7(6/9); (c) PU_{650}-7(2/9); (d) PU_{650}-7(0/9)

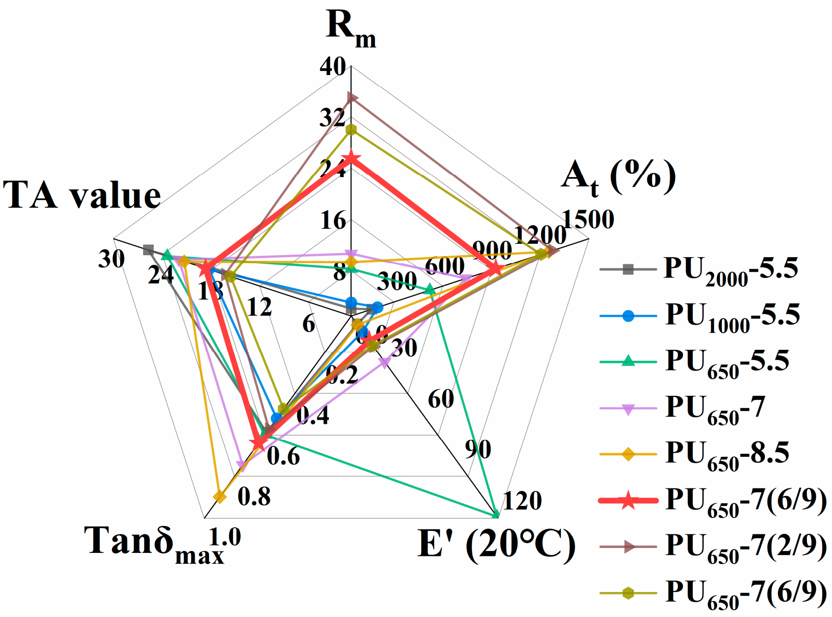

Figure S4 Radar plot of various PU matrix

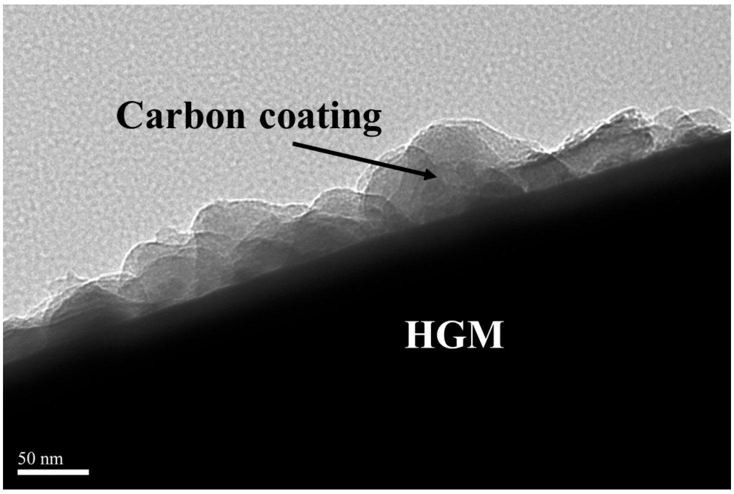

Figure S5 TEM image of HGM@C

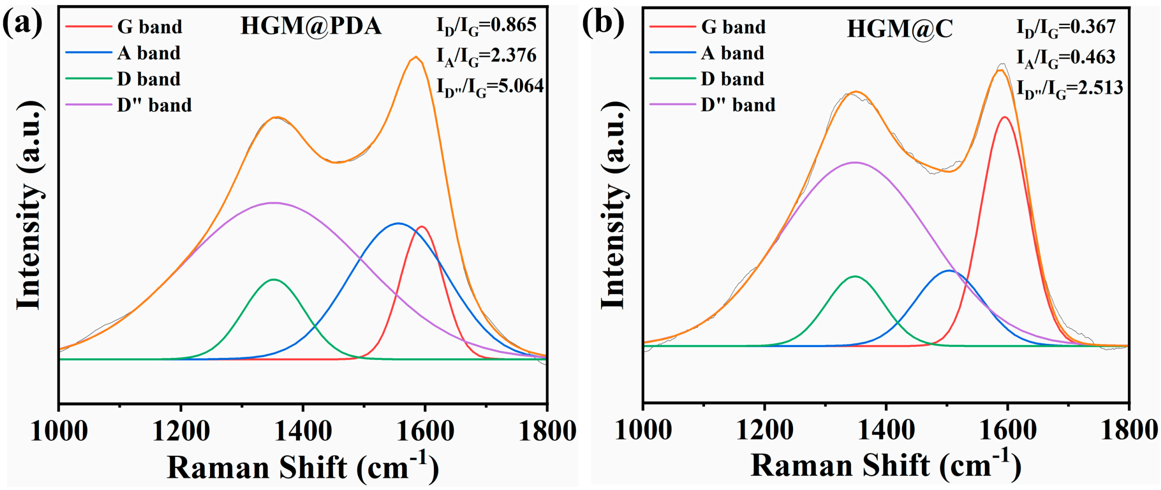

Figure S6 Raman peaks of D, G, A and D'' bands of (c) HGM@PDA; (d) HGM@C

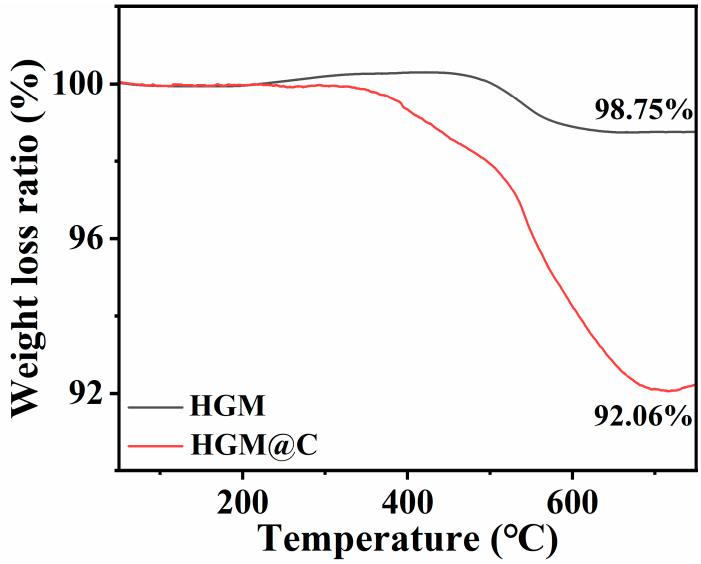

Figure S7 TG curves of HGM and HGM@C

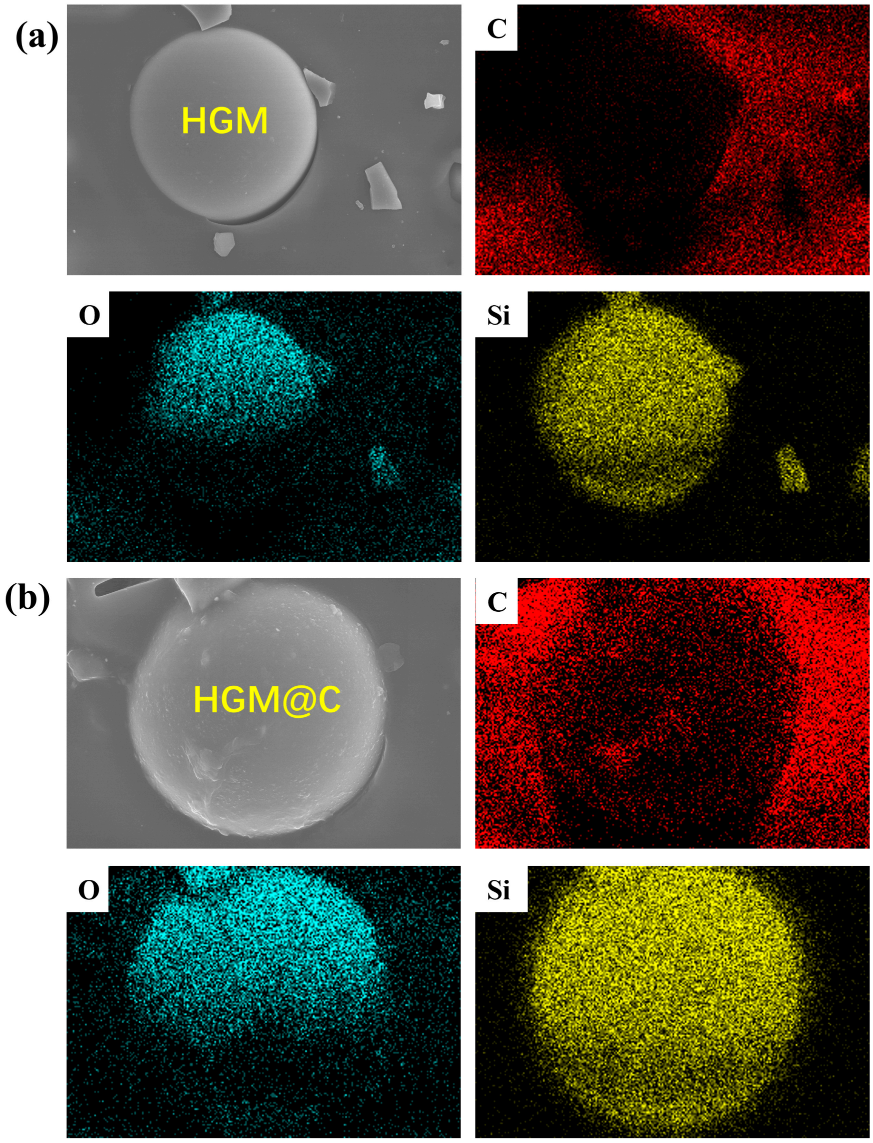

Figure S8 Distribution of element C, O, Si in debonding between PU and (a) HGM; (b) HGM@C
